# Leptin and IGF-1 in Infancy Are Associated With Variants in *DHCR7* and *CYP2R1* That Relate With Type 1 Diabetes and 25OHD

**DOI:** 10.1210/clinem/dgad263

**Published:** 2023-05-12

**Authors:** Antigoni Eleftheriou, Ken K Ong, Ieuan A Hughes, Clive J Petry

**Affiliations:** Department of Paediatrics, University of Cambridge, Cambridge CB2 0QQ, UK; Department of Paediatrics, University of Cambridge, Cambridge CB2 0QQ, UK; MRC Epidemiology Unit, University of Cambridge, Cambridge CB2 0QQ, UK; Institute of Metabolic Science, University of Cambridge, Cambridge CB2 0QQ, UK; Department of Paediatrics, University of Cambridge, Cambridge CB2 0QQ, UK; Department of Paediatrics, University of Cambridge, Cambridge CB2 0QQ, UK

**Keywords:** vitamin D, type 1 diabetes, leptin, IGF-1, early growth

## Abstract

**Context:**

Vitamin D has been variably implicated in risk of developing type 1 diabetes based on cohorts of at-risk individuals. Emergent type 1 diabetes in childhood is putatively preceded by altered growth.

**Objective:**

We explored whether polymorphisms in vitamin D metabolism genes modify risk of type 1 diabetes via effects on growth in a prospective, population-based cohort of infants.

**Methods:**

The Cambridge Baby Growth Study enrolled newborns from Cambridgeshire, UK, for follow-up in infancy. In 612 infants, we genotyped single nucleotide polymorphisms in vitamin D metabolism genes that relate with type 1 diabetes: rs10741657 and rs12794714 in *CYP2R1*, rs12785878 in *DHCR7*, and rs10877012 in *CYP27B1*. Multivariate linear regression analyses tested associations between genotypes and anthropometric indices (weight, length, and skinfold thickness) or growth-related hormones (C-peptide, IGF-1, and leptin) in infancy.

**Results:**

Birth weight showed borderline associations with the diabetes risk–increasing alleles in *CYP2R1*, rs10741657 (β = −.11, *P* = .02) and rs12794714 (β = −.09, *P* = .04). The risk-increasing allele rs12794714 was also associated with higher IGF-1 levels at age 24 months (β = .30, *P* = .01). At age 3 months, the risk-increasing allele rs12785878 in *DHCR7*, known to negatively associate with 25-hydroxyvitamin D levels, showed a positive association with leptin levels (β = .23, *P* = .009), which was pronounced in girls (*P* = .004) vs boys (*P* = .7).

**Conclusion:**

The vitamin D metabolism genes *DHCR7* and *CYP2R1* might influence infancy leptin and IGF-1 levels respectively. These findings open the possibility for a developmental role of vitamin D that is mediated by growth-related hormones with implications for the onset of type 1 diabetes autoimmunity.

Type 1 diabetes is a chronic disease that most often emerges in genetically susceptible children and adolescents following preclinical islet autoimmunity and destruction of insulin-secreting β-cells. Preclinical autoimmunity in type 1 diabetes is thought to develop by approximately 12 months of age when the immune system has matured enough to elicit an autopathogenic response (1). Vitamin D is believed to protect against autoimmunity owing to its nonclassical immunomodulatory action, which possibly involves suppression of effector Th1 or Th17 cells and preferential differentiation of regulatory T cells (2). Genetic studies of type 1 diabetes pinpointed susceptibility single nucleotide polymorphisms (SNPs) mapped to genes in the vitamin D metabolism milieu, namely synthesis (*DHCR7*) and hydroxylation (*CYP2R1, CYP27B1*) (3, 4). The diabetes risk–increasing alleles of some of these polymorphisms were found, by a genome-wide association study (GWAS), to correlate with lower levels of circulating 25-hydroxyvitamin D (25OHD) (5), the established indicator of vitamin D status.

The putative connection between vitamin D deficiency in early life and diagnosis of type 1 diabetes, or its prodrome of islet autoimmunity, provides the rationale for supplementation as a preventative strategy. This link tallies with epidemiological observations relating to the sunshine effect: the seasonality in diagnosis (lower in the summer than in autumn and winter) (6) and the north-south latitudinal gradient (7). Nevertheless, evidence of an association between risk of developing type 1 diabetes and vitamin D status at gestation or birth is lacking (8). Prospective cohort studies of at-risk children measured 25OHD levels in infancy and childhood and reported inconsistent findings: the Type 1 Diabetes Prediction and Prevention (DIPP) study (9) and the Diabetes Autoimmunity Study in the Young (DAISY) (10) independently reported lack of evidence, whereas The Environmental Determinants of Diabetes in the Young (TEDDY) study found an association between low serum 25OHD levels and increased risk of islet autoimmunity which was modified by a vitamin D receptor (VDR) genotype (11). Genetic studies have focused on *VDR*, which harbors many polymorphisms, but yielded divergent results about its predisposing influence on the development of type 1 diabetes (12).

Despite the checkered history relating vitamin D with diagnosis of diabetes in childhood, the Trial to Reduce IDDM [insulin-dependent diabetes mellitus] in the Genetically at Risk (TRIGR) study has more recently suggested that the involvement of vitamin D is traced in the early disease process, even before seroconversion, which points to its “developmental origin” (13). However, the authors of the TRIGR study cautioned about reverse causality, meaning that vitamin D deficiency might be the outcome of disease itself (13). It is, thus, of the essence to test for the objective effect of vitamin D on inherent risk of type 1 diabetes in a population that is not biased toward clinical disease.

Vitamin D reportedly enhances secretion of hormones, such as insulin (14), which is a critical growth factor in early life. Observational studies have suggested that risk of islet autoimmunity or type 1 diabetes increases with accelerated fetal growth, proxied by higher birth weight (15), or altered postnatal growth, measured as gains in weight (16, 17), or possibly length and adiposity (18). This line of evidence prompted us to investigate if the vitamin D metabolism milieu modifies inherent risk of type 1 diabetes via effects on growth and related hormones, namely C-peptide, used as a proxy for insulin; IGF-1, found predominantly conjugated with IGF binding protein-3 (IGFBP-3) from which it is liberated to bind to the IGF-1 receptor and promote linear growth (19); and leptin, which is a cytokine-like hormone produced in adipose tissue. Specifically, we aimed to explore associations between type 1 diabetes susceptibility polymorphisms mapped to vitamin D metabolism genes (*CYP2R1*, *DHCR7*, and *CYP27B1*) and anthropometric indices (weight, length, and skinfold thickness) or blood levels of endocrine regulators of growth (C-peptide, IGF-1, IGFBP-3, and leptin) in infancy. Our approach, which relies on (i) genetic loci rather than blood levels of vitamin D, and (ii) a cohort drawn from the general population vs enriched with at-risk children, addresses the confounding effect of emerging disease. On these grounds, our study holds the potential to shed light on pathways where vitamin D metabolism genes influence early endocrine processes that are pertinent to inherent risk of type 1 diabetes.

## Methods

### Study Design

We designed the current investigation based on using the Cambridge Baby Growth Study (CBGS), which is a prospective, observational birth cohort that recruited expectant mothers aged ≥16 years and their offspring during early pregnancy at the Rosie Maternity Hospital in Cambridgeshire, UK, between 2001 and 2009. The details of the CBGS were published elsewhere (20). A total of 1658 newborns were enrolled and followed up until the age of 2 years by trained pediatric research nurses. The growth of participating infants was monitored at a minimum of one study visit scheduled within 8 days of birth and at ages 3, 12, 18, and 24 months. Mode of infant feeding (breast milk only or formula milk) at age 3 months, maternal anthropometry, and sociodemographic data were captured by using structured questionnaires. The cohort is predominantly White Caucasian (>95%). Participation in the study was voluntary and all mothers gave written informed consent. The study was approved by the Cambridge local research ethics committee. The set of data included in the genotype-phenotype association study was based on a subcohort of 612 infants who had DNA available from cord blood or infancy capillary blood; the selection of samples to genotype was based on DNA availability as described previously (18).

### Phenotypes

#### Anthropometric indices

Infant anthropometric indices were measured at each study visit except for weight at birth which was captured from maternity hospital records. Weight was measured to the nearest 1 g using a Seca 757 electronic digital scale (Seca, UK). Supine length was measured to the nearest 0.1 cm using a Seca 416 infantometer (Seca, UK). Skinfold thickness was measured at 4 sites (triceps, subscapular, flank, and quadriceps) using a Holtain Tanner/Whitehouse Skinfold Caliper (Holtain, UK).

#### Blood sampling

Capillary blood was obtained from age 3 months onwards by heel prick (Tenderfoot, Elitech, UK) and partly stored at −20 °C until DNA extraction. The remaining sampled blood was blotted onto Whatman 903 untreated marker filter cards (Whatman, UK), air-dried at room temperature overnight, and stored at −20 °C.

#### DNA extraction

Genomic DNA was extracted by a chloroform-based method, quantified by Picogreen, and stored at 4 °C until genotyping.

#### Hormone assaying

Hormones were extracted from capillary dried blood spot (DBS) samples. C-peptide levels were measured using the Mercodia Ultrasensitive C-peptide ELISA for plasma (Mercodia Cat # 10-1141-01, RRID: AB_2819186) adapted for DBS, based on the adaptation of the Mercodia insulin assay for DBS (21). Circulating levels of IGF-1 and IGFBP-3 were measured using radioimmunoassays that had been respectively sourced from a manufacturer (Mediagnost, Tübingen, Germany) and developed in-house (22), for adaptation to DBS as described previously (23); the identical IGF-1 and similar IGFBP-3 immunoassays to those used are currently commercially available (Mediagnost Cat# R20, RRID: AB_2935798; Mediagnost, Cat # R11 RRID: AB_2935844, respectively). Leptin levels were determined by an in-house immunoassay using time-resolved fluorescence intensity (DELFIA) technology (PerkinElmer, MA, USA) and commercially available antibodies (R and D Systems Cat# MAB398, RRID: AB_2136056; R and D Systems Cat# BAM398, RRID: AB_2136057).

### Genotypes

#### SNP selection

We sourced type 1 diabetes susceptibility polymorphisms mapped to vitamin D metabolism genes from the study by Cooper et al (4), who analyzed a large number of samples from British case/controls in conjunction with families of European ancestry and identified SNPs in association with type 1 diabetes at the *P* ≤ .05 level of statistical significance: *CYP2R1*/11p15, rs10741657 (OR < 1, *P* = 3.0 × 10^−3^) and rs12794714 (OR > 1, *P* = 3.6 × 10^−3^); *DHCR7*/11q12, rs12785878 (OR > 1, *P* = 1.2 × 10^−3^); and *CYP27B1*/12q14, rs10877012 (OR < 1, *P* = 1.4 × 10^−4^), where “OR” denotes odds ratio for the minor allele and *P* is the combined level of statistical significance. Among these, the SNPs rs10741657 (*CYP2R1*) and rs12785878 (*DHCR7*) were associated with circulating 25OHD levels (*P* = 3.3 × 10^−20^ and *P* = 2.1 × 10^−27^, respectively) in a previous study among ∼30 000 European-descent individuals (5).

#### Genotyping and quality control

The SNPs were genotyped using the “Kompetitive Allele Specific PCR” (KASP) genotyping assay by LGC Genomics (Hoddesdon, UK). Quality control filters were applied upon excluding samples of reported poor DNA quality (n = 13). Call rates ranged from 95% to 97%. The Hardy-Weinberg equilibrium calculated from the goodness-of-fit chi-square test yielded *P* > .05 for each genetic variant. The SNPs were common, based on their respective allele and genotype frequencies (Table 1). We tested whether the selected SNPs are independent by assessing the extent of pairwise linkage disequilibrium (LD) (24); the SNPs in *CYP2R1* are in LD based on the D′ (1.0) but only moderately so based on the *r^2^* (0.5) All SNPs passed the quality control filters and were used in the association analyses.

**Table 1.** Type 1 diabetes-associated SNPs in vitamin D metabolism genes typed in the CBGS

Chromosome	Gene	SNP	Alleles	MAF	Genotype counts (frequencies)		
					Major	Minor	Hetero
11p15	*CYP2R1*	rs10741657	G > A	0.41	196 (34)	93 (16)	283 (49)
11p15	*CYP2R1*	rs12794714	G > A	0.40	207 (36)	96 (17)	278 (48)
11q12	*DHCR7*	rs12785878	T > G	0.24	338 (58)	37 (6)	205 (35)
12q14	*CYP27B1*	rs10877012	G > T	0.32	263 (45)	60 (10)	256 (44)

Abbreviations: Hetero, heterozygote; MAF, minor allele frequency; Major, major homozygote; Minor, minor homozygote.

### Calculations and Statistics

Weight and length were converted to age- and sex-appropriate SD scores (SDS), also corrected for gestational age for outcomes at birth and 3 months, based on the UK 1990 growth reference by using the LMS growth software (25). Measurements of skinfold thickness at each anatomical site were converted to internal SDS adjusted for age and sex, as well as gestational age for outcomes at birth and 3 months; an overall skinfold thickness index was calculated in each infant as an indicator of adiposity by taking the mean of SDS at the 4 sites as described previously (26).

The number of infants with genotype and anthropometric data was 597. Analysis was restricted to 586 infants, born between August 2001 and February 2009, after excluding those with extremes of growth trajectories (maternal type 1 diabetes, <36 weeks of gestation, and/or multifetal pregnancies) as described previously (18). Table 2 summarizes the availability of phenotypes by age at measurement, which reflects the attrition of measurements as the study timeline evolved. Anthropometric data were available from birth, whereas hormones were quantified from age 3 months, as heel pricking was not performed in neonates. We could not quantify C-peptide and leptin beyond the first year of life due to lack of available DBS samples, which had been prioritized for IGFs. Fewer than half of the infants included in the analysis had hormone levels measured at each study visit, which reflects the reduced consent/assent to heel pricking and insufficient DBS discs for assaying multiple hormones; nevertheless, the proportions of hormone data by age at measurement were similar to those in the entire CBGS cohort. Specifically, the percent of infants included in the analysis (n = 586) who had IGF-1 levels measured vs the respective percent in the CBGS cohort (n = 1658) were 36% vs 34% at 3 months, 28% vs 23% at 12 months, 23% vs 18% at 18 months, and 15% vs 10% at 24 months; comparative availability of IGFBP-3 measurements were similar to those for IGF-1 (18). C-peptide measurements were available in 21% of the subset analyzed vs 19% in the CBGS cohort at age 3 months, and 10% vs 9% respectively at age 12 months. The respective percentages for leptin levels were 21% vs 19% at age 3 months, and 16% vs 14% at age 12 months. Comparison of outcome measures by sex was performed by *t* test for anthropometric indices and Mann-Whitney U test for hormone levels.

**Table 2.** Counts (%) of infants with phenotype data by age at measurement (N = 586)

	Birth	3 months	12 months	18 months	24 months
Weight SDS	584 (100)	517 (88)	468 (80)	448 (76)	432 (74)
Length SDS	569 (97)	511 (87)	464 (79)	448 (76)	427 (73)
Skinfold SDS	567 (97)	517 (88)	466 (80)	448 (76)	434 (74)
C-peptide		123 (21)	56 (10)		
IGF-1		212 (36)	166 (28)	135 (23)	89 (15)
IGFBP-3		212 (36)	161 (27)	133 (23)	85 (15)
Leptin		121 (21)	96 (16)		

Abbreviations: IGF-1, insulin-like growth factor 1; IGFBP, IGF binding protein; SDS, SD score; Skinfold, skinfold thickness.

Association analyses between each phenotype (explained variable)—anthropometric index or circulating hormone levels—at each age measured, and SNP (explanatory variable) were performed by constructing parsimonious linear regression models with adjustment for preselected covariates. Additive genetic effects were assumed: 2 = two risk-increasing alleles, 1 = one risk-increasing allele, 0 = nil risk-increasing alleles, with the risk-increasing allele conferring risk of type 1 diabetes. Predictors to adjust for were determined *a priori* for each explained variable and age of infant in the CBGS cohort as described previously (18). Weight SDS and length SDS analyses were adjusted for maternal pre-pregnancy weight, maternal height, maternal smoking during pregnancy, parity, and mode of infant feeding at age 3 months. Skinfold thickness SDS models were adjusted for parity and mode of infant feeding at age 3 months. C-peptide models were adjusted for mode of infant feeding at age 3 months. IGF models were adjusted for sex and mode of infant feeding at age 3 months. Leptin models were adjusted for sex and parity.

Statistical analyses were performed using the IBM Statistical Package for Social Sciences software (IBM SPSS Statistics for Windows, Version 23.0; Armonk, New York, USA) and R (version 3.3.2; R Foundation for Statistical Computing, Vienna, Austria). Tests were two-tailed and a *P* < .05 was considered statistically significant. Corrections for multiple comparisons were not performed, as this was an exploratory study whose outcomes were intercorrelated.

## Results

A total of 586 infants (315 boys and 271 girls) with genetic and anthropometric data were included in the genotype-phenotype association study, a proportion of whom also had IGF levels measured as described previously (18). Table 3 displays the descriptive statistics of hormones that were additionally quantified in these infants and used for the current investigation. Whereas C-peptide levels were on average similar by sex, mean leptin levels were elevated in girls compared with boys and approximately halved from age 3 to 12 months for both sexes. Considering that sample sizes were small, we confirmed that these patterns for hormone levels by sex and age at measurement were in accord with those in the CBGS cohort (data not shown).

**Table 3.** C-peptide and leptin levels (mean, SD) stratified by sex and age at measurement (N = 586)

	Boys		Girls		
	n	Mean ± SD	n	Mean ± SD	*P*
3 months					
C-peptide (pmol/L)	63	702 ± 386	60	665 ± 426	.3
Leptin (ng/mL)	60	2.0 ± 1.1	61	3.4 ± 2.0	<.001
12 months					
C-peptide (pmol/L)	36	602 ± 344	20	656 ± 364	.4
Leptin (ng/mL)	56	1.1 ± 0.6	40	1.6 ± 1.1	.009

Summary statistics (β, *P* value) of regression analyses of type 1 diabetes genetic risk at each locus—proxied by the sum of diabetes risk–increasing alleles—on standardized anthropometric indices or circulating hormone levels are displayed in Tables 4 and 5 respectively. Both SNPs in *CYP2R1* were nominally associated with lower birth weight, namely rs10741657 (β = −.11, *P* = .02) and rs12794714 (β = −.09, *P* = .04). The SNP rs12794714 was weakly associated with shorter length at age 18 months (β = −.11, *P* = .03), but higher IGF-1 (β = .30, *P* = .01) and IGFBP-3 (β = .24, *P* = .04) levels at age 24 months. That the positive association with IGF-1, a regulator of linear growth, was curiously preceded by a negative association with length SDS was explained by a decline in the linear growth trajectory at age 18 months, which was also observed in the entire cohort and attributed to the enrichment of the CBGS with breastfed children vs the reference population. Nevertheless, the relative magnitude of IGF-1 levels and length SDS by genotype rs12794714 mirrored each other in late infancy, with carriers of 2 risk-increasing alleles having on average the lowest levels at age 18 months but catching up and overtaking heterozygotes thereafter (data not shown).

**Table 4.** Linear multivariate regression models of type 1 diabetes susceptibility SNPs in vitamin D metabolism genes on anthropometric indices by age at measurement

			Birth		3 months		12 months		18 months		24 months	
	Gene	SNP	β	*P*	β	*P*	β	*P*	β	*P*	β	*P*
Weight SDS												
*CYP2R1*		rs10741657	−.11*^a^*	.02	.02	.7	.01	>.9	.00	>.9	−.02	.6
*CYP2R1*		rs12794714	−.09*^a^*	.04	−.03	.6	−.01	.9	−.04	.4	−.04	.4
*DHCR7*		rs12785878	−.01	.8	−.01	.8	−.10	.05	−.09	.1	−.07	.2
*CYP27B1*		rs10877012	.04	.4	−.01	.8	−.01	.9	−.02	.7	−.04	.5
Length SDS												
*CYP2R1*		rs10741657	−.09	.06	−.04	.4	−.04	.4	−.07	.2	−.02	.7
*CYP2R1*		rs12794714	−.02	.6	−.05	.3	−.03	.5	−.11*^a^*	.03	−.07	.2
*DHCR7*		rs12785878	−.05	.3	−.09	.1	−.05	.3	−.07	.2	−.08	.1
*CYP27B1*		rs10877012	−.01	.9	−.01	.9	−.04	.4	−.02	.6	−.02	.7
Skinfold SDS												
*CYP2R1*		rs10741657	.01	.8	.01	.8	.03	.6	.05	.4	.05	.3
*CYP2R1*		rs12794714	.03	.5	−.06	.2	.00	>.9	−.01	.9	.04	.5
*DHCR7*		rs12785878	.03	.5	−.01	.8	−.04	.4	−.06	.3	.01	.9
*CYP27B1*		rs10877012	−.01	.8	−.05	.3	.00	>.9	−.04	.4	−.06	.2

Regression coefficients are displayed. Weight SDS and length SDS models were adjusted for maternal pre-pregnancy weight, maternal height, maternal smoking during pregnancy (yes/no) for outcomes at birth only, parity (primiparous, yes/no) for outcomes at birth and age 3 months only, and mode of infant feeding (breast milk only at age 3 months, yes/no) except for outcomes at birth. Skinfold thickness SDS models were adjusted for parity (primiparous, yes/no) for outcomes at birth and age 3 months only, and mode of infant feeding (breast milk only at age 3 months, yes/no) except for outcomes at birth.

Abbreviations: SDS, SD score; Skinfold, skinfold thickness; SNP, single nucleotide polymorphism.

^*a*^ *P* < .05

**Table 5.** Linear multivariate regression models of type 1 diabetes susceptibility SNPs in vitamin D metabolism genes on blood hormone levels by age at measurement

			3 months		12 months		18 months		24 months	
Gene		SNP	β	*P*	β	*P*	β	*P*	β	*P*
C-peptide (pmol/L)										
*CYP2R1*		rs10741657	.04	.7	−.11	.4				
*CYP2R1*		rs12794714	.06	.5	−.18	.2				
*DHCR7*		rs12785878	−.01	.9	.11	.5				
*CYP27B1*		rs10877012	−.02	.8	−.16	.3				
IGF-1 (ng/mL)										
*CYP2R1*		rs10741657	−.00	>.9	.09	.3	.14	.1	.16	.2
*CYP2R1*		rs12794714	.07	.3	−.04	.6	−.03	.7	.30*^a^*	.01
*DHCR7*		rs12785878	.06	.4	.03	.7	.06	.5	.06	.6
*CYP27B1*		rs10877012	.07	.4	.03	.7	−.03	.7	.15	.2
IGFBP-3 (ng/mL)										
*CYP2R1*		rs10741657	−.14	.06	−.01	.9	−.08	.4	.08	.5
*CYP2R1*		rs12794714	−.04	.6	−.01	.9	−.07	.5	.24*^a^*	.04
*DHCR7*		rs12785878	.03	.7	.02	.8	−.03	.8	.15	.2
*CYP27B1*		rs10877012	−.03	.7	.04	.7	−.05	.5	.20	.09
Leptin (ng/mL)										
*CYP2R1*		rs10741657	.12	.2	.14	.2				
*CYP2R1*		rs12794714	.16	.1	.21*^a^*	.04				
*DHCR7*		rs12785878	.23*^b^*	.009	.02	.8				
*CYP27B1*		rs10877012	.11	.2	−.04	.7				

Regression coefficients are displayed. C-peptide models were adjusted for mode of infant feeding (breast milk only at age 3 months, yes/no). IGF models were adjusted for sex and mode of infant feeding (breast milk only at age 3 months, yes/no). Leptin models were adjusted for sex and parity (primiparous, yes/no).

Abbreviations: IGF-1, insulin-like growth factor 1; IGFBP, IGF binding protein; SNP, single nucleotide polymorphism.

^*a*^ *P* < .05.

^*b*^ *P* < .01.

**Figure 1.**
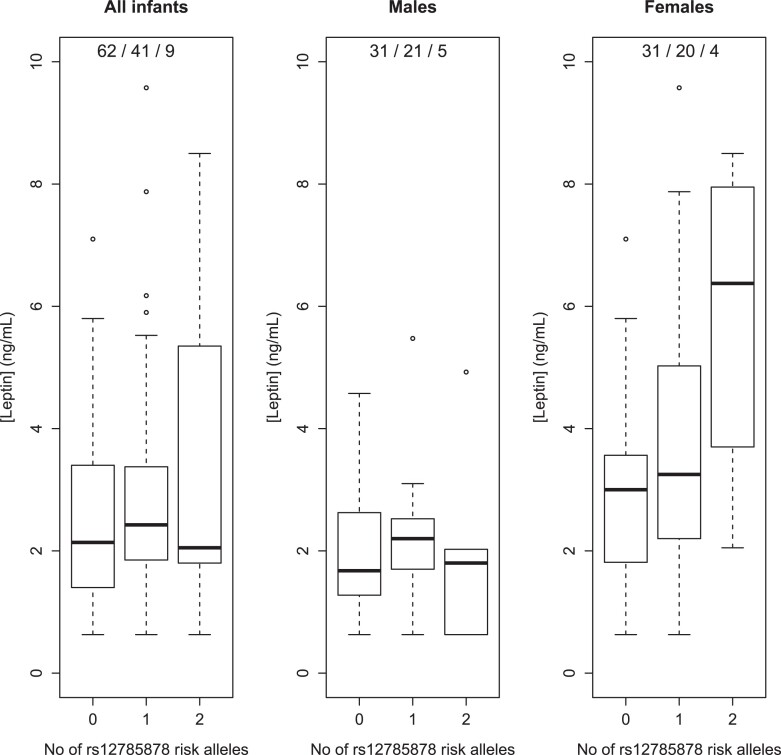
Leptin levels at age 3 months by rs12785878 genotype: TT, GT, GG, where G is the diabetes risk–increasing allele. Genotype frequencies are listed in legends.

The SNP rs12785878 in *DHCR7* was positively associated with leptin levels at age 3 months (β = .23, *P* = .009). Specifically, mean leptin levels increased from 2.4 to 3.0 to 3.7 ng/mL in carriers of 0, 1, and 2 risk-increasing alleles rs12785878 respectively. Sex-specific analysis showed that this association was evident in girls (β = .38, *P* = .004) but not in boys (*P* = .7) (Fig. 1), which was confirmed by testing for the SNP-sex interaction term (β = .68, *P* = .01). Mean leptin levels in boys were similar by rs12785878 genotype, whereas in girls they showed an increase from 2.9 to 3.8 to 5.8 ng/mL by addition of a risk-increasing allele rs12785878, with the caveat that sample sizes were small, particularly, for homozygotes for the risk-increasing allele. There was no evidence of an association between the variant in *CYP27B1* and growth. Among phenotypes, neither skinfold thickness nor C-peptide levels showed significant associations.

## Discussion

This is the first study, to the best of our knowledge, to investigate differential associations between polymorphisms mapped to vitamin D metabolism genes that have been known to associate with risk of type 1 diabetes (*CYP2R1*, *DHCR7*, and *CYP27B1*) (4) on early anthropometry (weight, length, and skinfold thickness) or blood levels of growth-related hormones (C-peptide, IGF-1, and leptin) in a general population-based cohort of infants. At birth, the diabetes risk–increasing polymorphisms in *CYP2R1*, which encodes a hepatic microsomal enzyme, were weakly associated with lower weight. One of these was also associated with IGF-1 and IGFBP-3 levels in late infancy. At age 3 months, the risk-increasing variant in *DHCR7*, which encodes the enzyme in cholesterol and vitamin D biosynthesis, was associated with higher leptin levels. There was no evidence of a relationship between early growth and the intronic variant in *CYP27B1* (the only SNP in the vitamin D metabolism milieu that is genome-wide significant for risk of type 1 diabetes), suggesting that the predominantly renal enzyme 1α-hydroxylase is unlikely implicated in growth-related pathways. Similarly, C-peptide levels did not yield significant associations, which could be attributed to methodological factors, eg, low statistical power, or blood sampling at random time points and without consideration of timing relative to infant feeds (eg, non-fasted). Alternatively, the null results might be explained by the likely absence of a biological pathway between insulin and vitamin D. Contrary to observational data, intervention studies revealed a lack of effect of vitamin D on insulin levels in healthy (27) or overweight/obese (28) individuals, raising the possibility that confounders explain the association that underpinned our hypothesis. Nevertheless, the genotype-hormone associations should be interpreted cautiously because of small samples analyzed. Further, mathematical corrections were not made for multiple comparisons of highly correlated outcomes. Assessing the influence of uncorrected multiple testing, 5% of tests are expected to produce a *P* value of less than .05 due to random variation; in our study, 6% of tests gave a *P* value below the threshold, suggesting that some statistically significant associations may be false positives and require further evidence, that is, replication in larger cohorts, to confirm that they are biologically significant. Herein, we interrogate their biological plausibility based on existing knowledge.

The diabetes risk–increasing alleles in *CYP2R1* (rs10741657 and rs12794714) showed borderline associations with lower birth weight. The risk-increasing allele rs10741657 was previously associated with lower concentration of circulating 25OHD (5). Neonatal 25OHD levels are reportedly in an inverted U-shaped association with birth weight (29). Our finding pinpoints *CYP2R1*, which is expressed in the liver, as a possible contributor to the relationship between 25OHD and fetal growth; rs12785878 in *DHCR7*, which is also genome-wide significant for 25OHD levels (5), did not associate with birth weight. Even though the association found here does not corroborate the link between higher birth weight and risk of type 1 diabetes, the results imply that the involvement of 25OHD in early growth pathways is mediated by the hepatic enzyme 25-hydroxylase, which possibly masks the action of a hepatic growth factor. That rs12794714 was associated with IGF-1 and IGFBP-3 levels at age 24 months reinforces the possibility of a molecular interaction between 25-hydroxylase and IGFs, aligning with our prior findings about the high-risk HLA-DR3, which underscored the interaction between IGF-1 and the immune system (18).

Our most important finding was that progressive addition of the diabetes risk–increasing (4), and 25OHD-lowering (5), allele rs12785878 mapped to *DHCR7* was associated with higher leptin levels at age 3 months in an allele-dependent manner (addition of each risk-increasing allele increased mean leptin levels by ∼0.65 ng/mL). *DHCR7* encodes 7-dehydrocholesterol reductase (DHCR7), the enzyme which acts as a “switch” in the cholesterol and vitamin D biosynthesis in the Kandutsch-Russell pathway, whereby degradation of DHCR7, and ensuing accumulation of substrate 7-dehydrocholesterol, increases vitamin D production (30). An inverse relationship between 25OHD and leptin levels was established by a meta-analysis of observational studies, albeit not confirmed by intervention studies (31), which concurs with the association found here in the context of prior knowledge, ie, allele rs12785878 is associated negatively with 25OHD (5) and positively with leptin levels. Our finding suggests that *DHCR7* is a determinant of the link between 25OHD and leptin, which implies pleiotropy (explaining the discordance between studies), or hints to the biological pathway linking the 2 hormones.

The association between rs12785878 and leptin levels was unexpectedly pronounced in girls vs boys, which is suggestive of the involvement of an endocrine factor of differential concentrations between males and females, or a sex steroid (eg, testosterone and estradiol). Female sex hormones were not measured in the CBGS. Nevertheless, evidence suggests a crosstalk between leptin and sex hormones. Leptin receptors are found in the human pituitary, which might explain how leptin influences gonadotropin release, while its own secretion is regulated by sex hormones (32). In juvenile female rats, leptin induces pituitary cells to synthesize and secrete gonadotropins independent of hypothalamic GnRH (33). In the context of literature, there is ground to postulate that our finding paves the way for a newfound, sex-dimorphic role of *DHCR7*, mediated by leptin, in the postnatal window of pituitary activation, which influences development of adaptive immunity. The transient activation of the hypothalamic-pituitary-gonadal axis along the first 6 months of life is known as minipuberty and is characterized by a rise in gonadotropins and sex hormones, similar to adult levels (34). Moreira-Filho et al (35) contended that minipuberty is a period when sex steroids act on the thymus (the gland of T-cell central tolerance) based on their finding that global gene expression was downregulated in thymic tissue of female vs male infants younger than 6 months, which was conceivably mediated by the estrogen surge since the effect disappeared later in infancy. In the CBGS, the association between rs12785878 and leptin levels was found at age 3 months, which is around the time in neonatal life when levels of gonadotropins putatively peak (34). Evidence of an association between leptin measurement and rs12785878 is currently absent from the National Human Genome Research Institute-European Bioinformatics Institute (NHGRI-EBI) GWAS catalogue (36). This suggests that the effect size of the association found here does not achieve genome-wide significance even in larger populations, or the SNP was not directly genotyped in prior studies, or, possibly, the association is relevant to neonatal life of development significance.

Drawing similarities of vitamin D biosynthesis between humans and simpler organisms lends credence to a development role. 7-Dehydrocholesterol is the precursor of ecdysteroid, a steroid hormone that is metabolized by cytochrome P450 enzymes and plays essential roles in developmental transitions at specific times throughout the life course of insects (37). Ecdysteroid influences development by stimulating secretion of IGF-like peptides in insect body fat, which is the equivalent of liver and adipocytes in vertebrates (38). This opens the possibility of similar molecular interactions between vitamin D precursor/metabolites and liver- or adipose-derived peptides that could solve the puzzle of the putative link between vitamin D status and early growth in humans.

The first months of life present an apt window for probing into inherent alterations of physiology, owing to the dominance of genetic factors and reduced confounding effects related to lifestyle and the environment. On these grounds, the CBGS lent itself to our investigation because it provides access to frequent measurements in early life of an ethnically homogeneous population; however, generalization to other populations becomes limited. Our approach to tap into a general population-based cohort and investigate genetic variants in the vitamin D metabolism milieu was instrumental for teasing out confounders of hypovitaminosis D and sign-posting unbiased mechanisms. In doing so, we were limited by the small count of infants with hormone measurements and the absence of these within the first 3 months of life, which is a window of the fastest endocrine changes. Furthermore, all but one of the SNPs investigated here are not genome-wide significant for risk of type 1 diabetes; however, the lower effect size could be partially explained by a sex-dimorphic influence that was not accounted for by genetic studies (unlike most autoimmune diseases which are female-predominant, type 1 diabetes affects males and females almost equally).

## Conclusion

The associations found between type 1 diabetes susceptibility variants in vitamin D metabolism genes and leptin or IGF-1 levels in infancy convey a possible developmental role of the vitamin D metabolism milieu, which might set off a chain of events in the first months of life that trigger intertwined endocrine and immune changes with effects on self-tolerance and risk of type 1 diabetes. Our findings may have implications for genetically similar diseases that manifest later in life when hormones surge again. A Mendelian randomization analysis suggested a causal pathway between the 25OHD-lowering allele rs12785878 (*DHCR7*) and increased risk of multiple sclerosis (39), which is a Th1-dominant disease. A question that arises is whether leptin mediates this reported relationship. In addition to its established metabolic regulatory effect, leptin stimulates Th1 response and suppresses regulatory T-cell expansion (40).

Future observational studies should seek to replicate the genotype-hormone associations found here and query their sex dimorphism at other ages in infancy, childhood, and adulthood, as well as in other ethnic populations. In addition, our main result could guide experimental studies in the direction of identifying mechanisms linking DHCR7 (and milieu), leptin, and possibly, estrogen which could aid our understanding of the precise role of vitamin D in the origins and development of autoimmune diabetes.

## Data Availability

Original data generated and analyzed during this study, with the exception of the genotypes for ethical and consensual reasons, are available at https://doi.org/10.17863/CAM.96591.

## Abbreviations

25OHD: 25-hydroxyvitamin D
CBGS: Cambridge Baby Growth Study
DBS: dried blood spot
DHCR7: 7-dehydrocholesterol reductase
GWAS: genome-wide association study
IGF-1: insulin-like growth factor 1
IGFBP: IGF binding protein
LD: linkage disequilibrium
MAF: minor allele frequency
OR: odds ratio
SDS: SD score
SNP: single nucleotide polymorphism
